# 

**Published:** 1889-05

**Authors:** 


CONTENTS.

Vol. 36 No. 5.

| | PAGE. |
|---|---|
| And Now Comes Waterville | 97 |
| Health and Hell | 100 |
| How Women Rest | 103 |
| Cinnamon | 104 |
| Water in Relation to Obesity | 104 |
| Uses of Cotton Seed | 105 |
| What Is Heredity | 106 |
| Valuable Remedies in Diphtheria | 107 |
| Execution by Electricity | 108 |
| Origin of Strong Liquors | 109 |
| Lard—Its Adulteration, etc | 110 |
| The Consumption of Opium, in China | 111 |
| Christian Science Outdone | 112 |
| Sleep | 112 |
| The True Relations of Filth to Diphtheria | 114 |
| A Safe Cordial | 114 |
| Jessie K., on Kissing | 115 |
| An Appreciative Husband | 115 |
| Dress | 115 |
| Fruit as Food | 116 |
| Knitted Dish Cloths | 116 |
| Book Notices | 117 |
| Household | 118 |
| Miscellaneous | 119 |
| A Little Poet | 120 |

INDEX TO ADVERTISEMENTS OF HALF A PAGE AND OVER.

| | PAGE. |
|---|---|
| Murdock's Liquid Food Co | I |
| Esoteric Publishing Co | II |
| Celluloid | II |
| E. C. Morris & Co | IV |
| Lactopeptine | V |
| Hollow Suppositories | VI |
| Revised Premium List | VIII |
| Rio Chemical Co | X |
| The Herb Cure Co | XI |

